# Supplementary figures and images for: Modular and cloud-based bioinformatics pipelines for high-confidence biomarker detection in cancer immunotherapy clinical trials
Source: PLoS One. 2025 Aug 26;20(8):e0330827. doi: 10.1371/journal.pone.0330827 (PMC12380322; doi:10.1371/journal.pone.0330827)

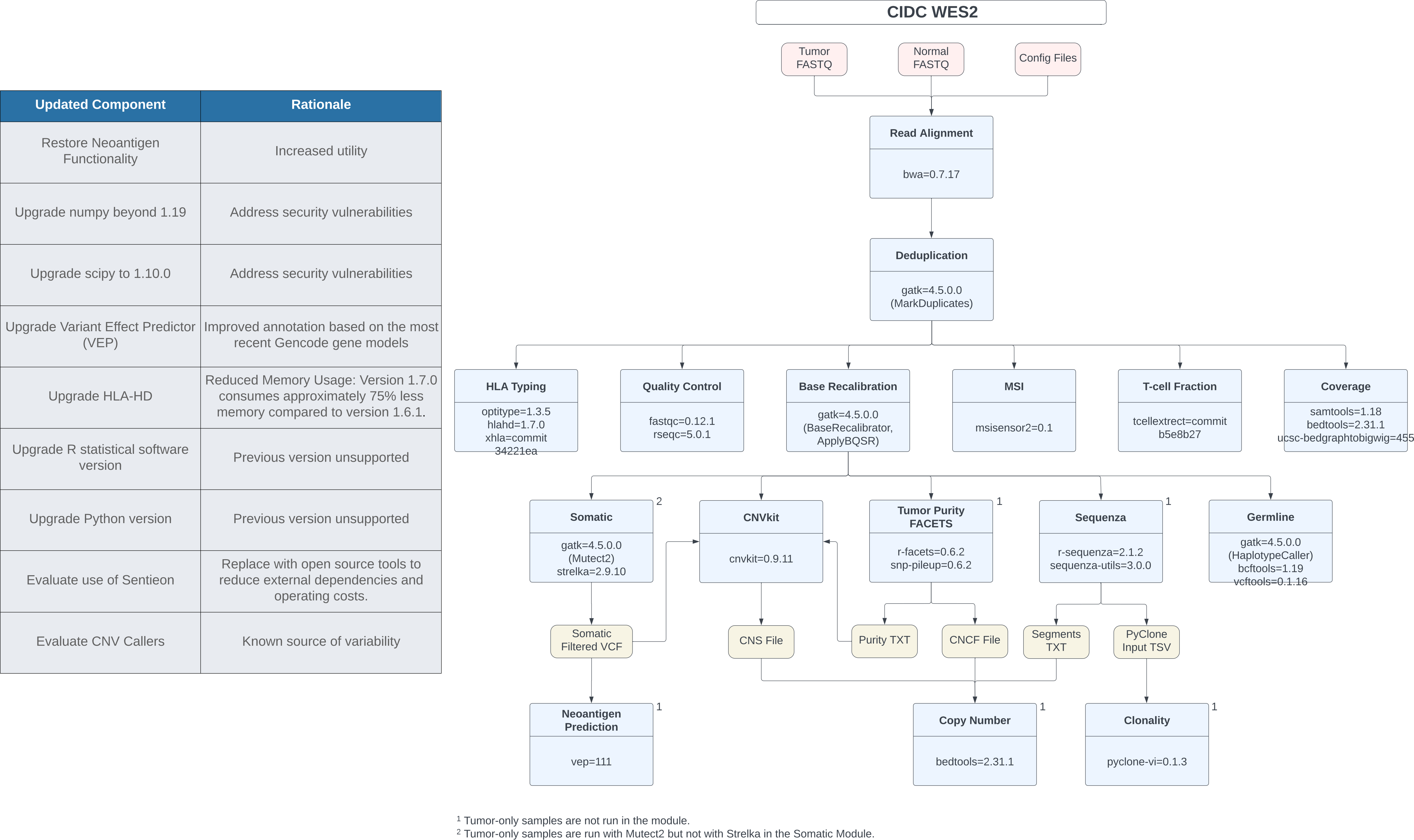

Supplement: S1 Fig — (left) Specific pipeline changes associated with the enhanced pipeline and the rationale for the changes are listed. (right) Workflow with the key software and software versions associated with each step shown. (TIF) [file pone.0330827.s001.tif]

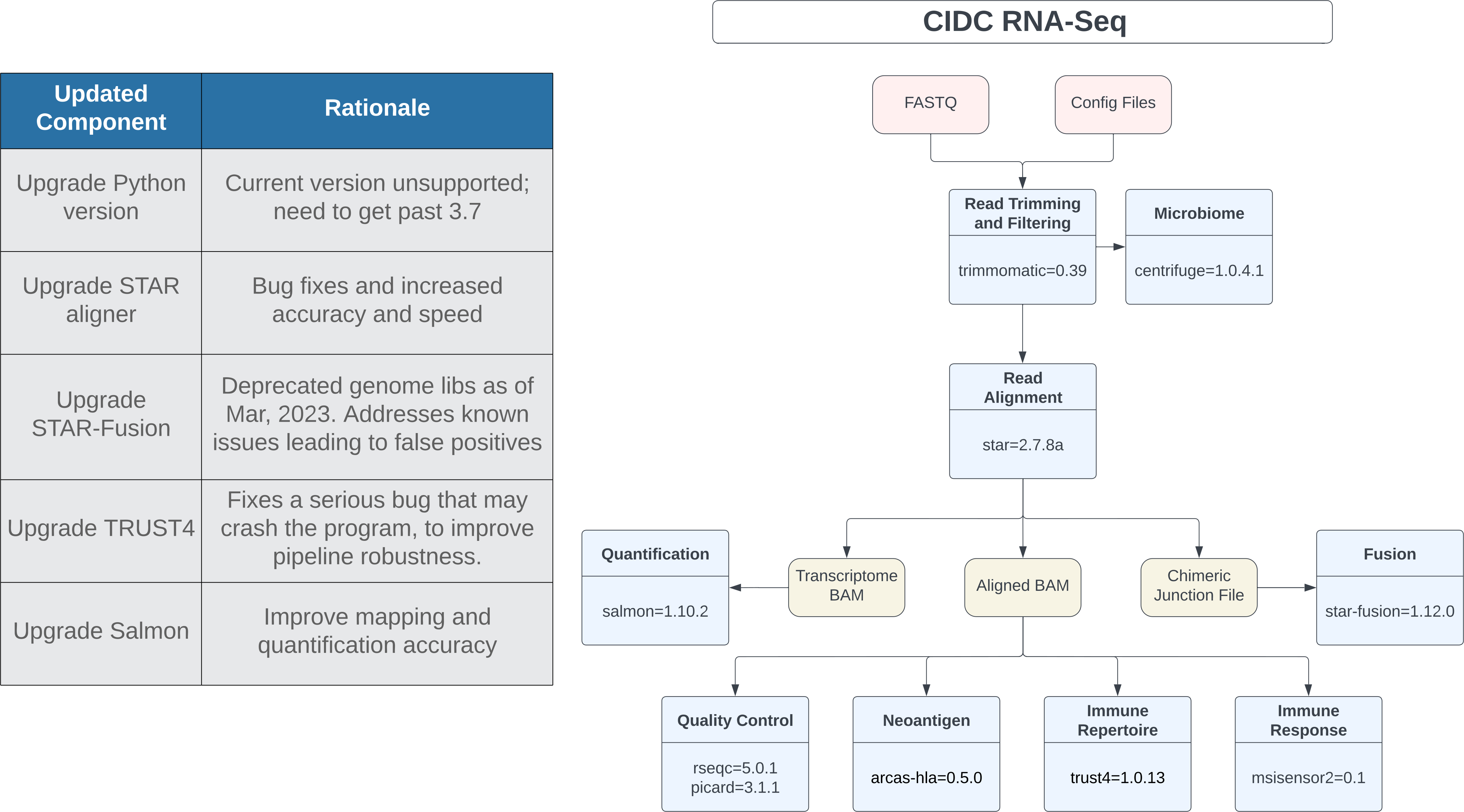

Supplement: S2 Fig — (left) Specific pipeline changes associated with the enhanced pipeline and the rationale for the changes are listed. (right) Workflow with the key software and software versions associated with each step shown. (TIF) [file pone.0330827.s002.tif]

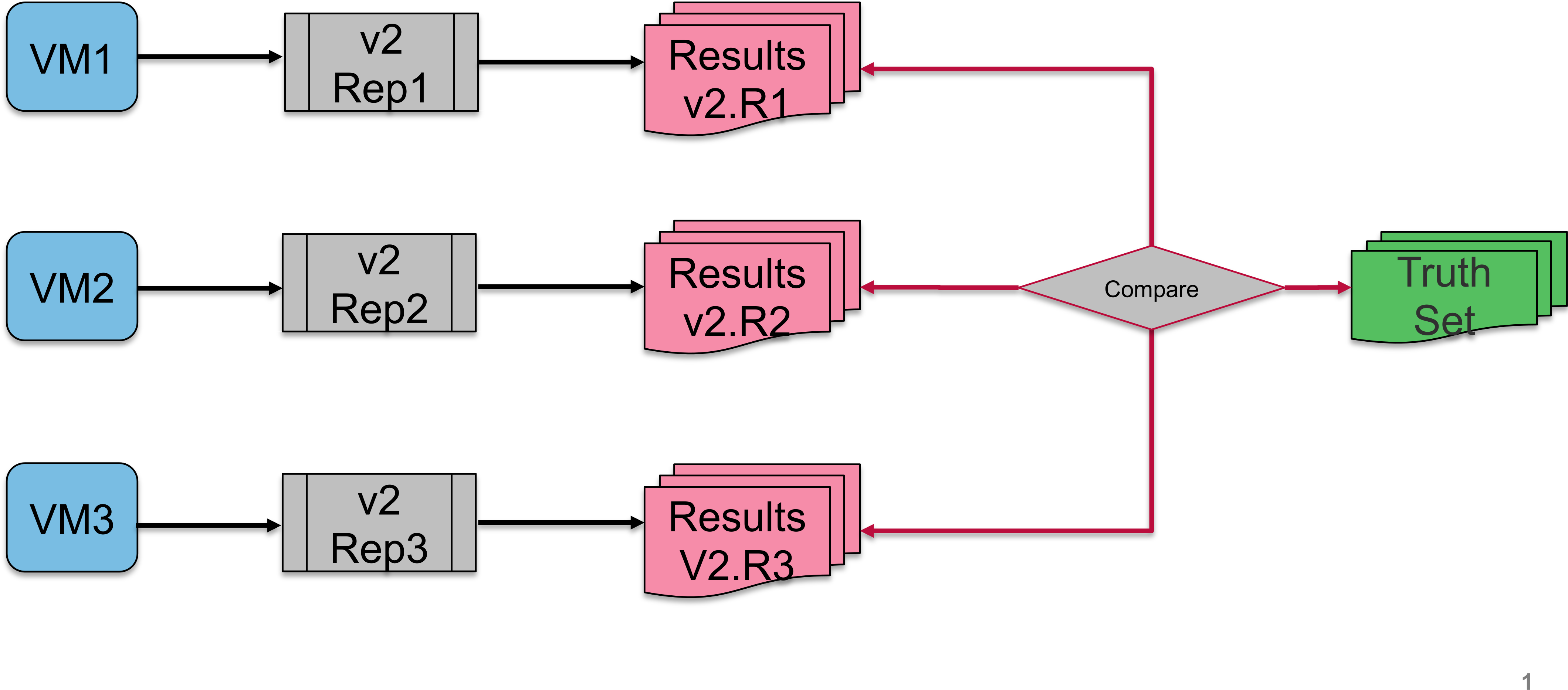

Supplement: S3 Fig — All Validation runs were experimental replicates, with a unique user and virtual machine (VM) being used for each run. In total, the validation was performed in triplicate to determine intra-pipeline reproducibility. The output of the triplicate runs was also compared pre-selected truth sets to measure precision and recall, along with other metrics. (TIF) [file pone.0330827.s003.tif]

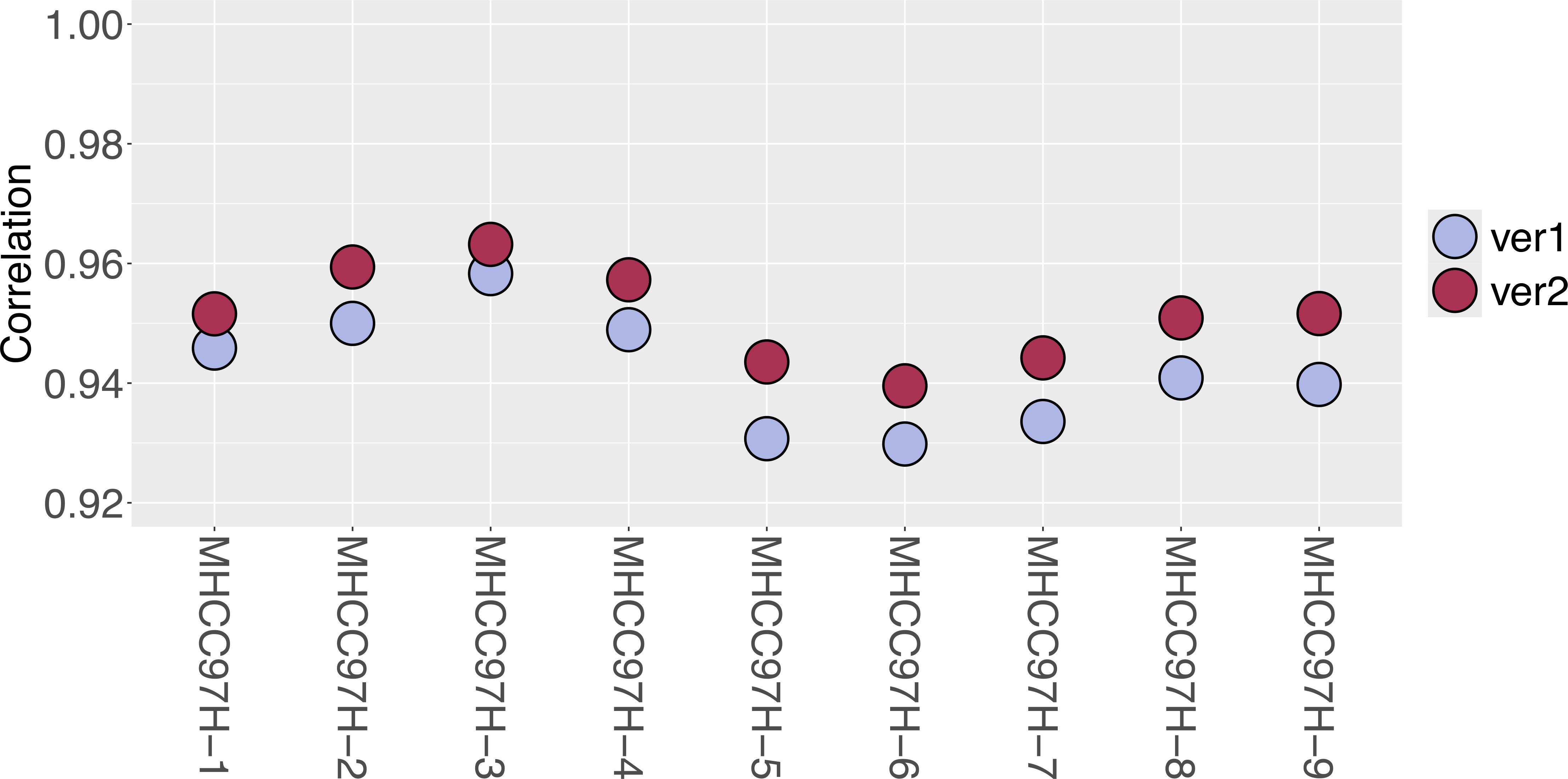

Supplement: S4 Fig — Comparative analysis of transcript quantification metrics between the original and enhanced RNA-Seq pipelines. The supplemental analysis includes evaluation of samples described in Lu S, et al. [12]. (TIF) [file pone.0330827.s004.tif]
