# Supplementary material for: Modular and cloud-based bioinformatics pipelines for high-confidence biomarker detection in cancer immunotherapy clinical trials
Source: PLoS One. 2025 Aug 26;20(8):e0330827. doi: 10.1371/journal.pone.0330827 (PMC12380322; doi:10.1371/journal.pone.0330827)
Supplement: Attachment — Submitted filename: Response to Reviewers.docx [file pone.0330827.s010.docx]

**Response to Reviewers**

07/16/2025

Dear Surya Saha,

We would like to thank you for your careful consideration of our manuscript. We have reviewed the points that you and the reviewers have raised and have modified the manuscript accordingly. We appreciate your feedback, as we think it has helped to improve the manuscript. Below, please find our point-by-point responses:

## From the Academic Editor

1. Please ensure that your manuscript meets PLOS ONE's style requirements, including those for file naming. The PLOS ONE style templates can be found at

<https://journals.plos.org/plosone/s/file?id=wjVg/PLOSOne_formatting_sample_main_body.pdf> and [https://journals.plos.org/plosone/s/file?id=ba62/PLOSOne_formatting_sample_title_authors_affiliations.pdf](https://gcc02.safelinks.protection.outlook.com/?url=https%3A%2F%2Ftrack.editorialmanager.com%2FCL0%2Fhttps%3A%252F%252Fjournals.plos.org%252Fplosone%252Fs%252Ffile%253Fid%3Dba62%252FPLOSOne_formatting_sample_title_authors_affiliations.pdf%2F1%2F010f01975dc23529-002f8f72-4384-4fed-a3be-ed8a7c40519b-000000%2FzFLPX2Fsbz35rjfPHuEHGd99neBD7WgQ07GJv3673bs%3D215&data=05%7C02%7Cnick.renzette%40nih.gov%7Cd2d75bd7fad64e1d1c2608dda8b46af1%7C14b77578977342d58507251ca2dc2b06%7C0%7C0%7C638852215328303886%7CUnknown%7CTWFpbGZsb3d8eyJFbXB0eU1hcGkiOnRydWUsIlYiOiIwLjAuMDAwMCIsIlAiOiJXaW4zMiIsIkFOIjoiTWFpbCIsIldUIjoyfQ%3D%3D%7C0%7C%7C%7C&sdata=CiExFFEoWj7bvAYlYHiYhXKzvTdEp8E1E1Xrdz0fZLs%3D&reserved=0)

**Response:** We have reformatted the manuscript, tables and figures to match PLOS ONE’s style requirements.

2. Thank you for stating the following financial disclosure:

[Funder Name: National Cancer Institute

Grant ID: 140D0421F0589].

Please state what role the funders took in the study.  If the funders had no role, please state: "The funders had no role in study design, data collection and analysis, decision to publish, or preparation of the manuscript."

If this statement is not correct you must amend it as needed.

Please include this amended Role of Funder statement in your cover letter; we will change the online submission form on your behalf.

**Response:** The funders had no role in study design, data collection and analysis, decision to publish, or preparation of the manuscript. Our cover letter has been updated with this information and we appreciate you updating the submission on our behalf.

3. Thank you for stating the following in your manuscript:

[Additional support is made possible through the NCI Clinical Reporting and Research Informatics (CRRI) Support Services Contract 140D0421F0589 (Essex Management)]

We note that you have provided funding information that is currently declared in your Funding Statement. However, funding information should not appear in the Acknowledgments section or other areas of your manuscript. We will only publish funding information present in the Funding Statement section of the online submission form.

Please remove any funding-related text from the manuscript and let us know how you would like to update your Funding Statement. Currently, your Funding Statement reads as follows:

[Funder Name: National Cancer Institute

Grant ID: 140D0421F0589].

Please include your amended statements within your cover letter; we will change the online submission form on your behalf.

**Response:** We have removed funding information from the Acknowledgements section. Our funding statement should read:

Scientific and financial support for the Cancer Immune Monitoring and Analysis Centers-Cancer Immunologic Data Center (CIMAC-CIDC) Network are provided through the National Cancer Institute (NCI) through NCI contract 140D0421D0007 to the CIDC operated by NCI. Additional support is made possible through the NCI Clinical Reporting and Research Informatics (CRRI) Support Services Contract 140D0421F0589 (Essex Management).

Additional financial support for the CIMAC-CIDC Network is provided by the Partnership for Accelerating Cancer Therapies (PACT) public-private partnership (PPP) made possible through funding provided to the Foundation for the National Institutes of Health (FNIH) by: AbbVie Inc., Amgen Inc., Boehringer-Ingelheim Pharma GmbH & Co. KG., Bristol-Myers Squibb, Celgene Corporation, Genentech Inc, Gilead, GlaxoSmithKline plc, Janssen Pharmaceutical Companies of Johnson & Johnson, Novartis Institutes for Biomedical Research, Pfizer Inc., and Sanofi.

Our cover letter has been updated this information.

4. We notice that your supplementary figures and tables are included in the manuscript file. Please remove them and upload them with the file type 'Supporting Information'. Please ensure that each Supporting Information file has a legend listed in the manuscript after the references list.

**Response:** We have removed the supplementary figures and tables and saved them as separate ‘Supporting Information’ files. The associated legends are included in the manuscript after the reference list.

5. Please review your reference list to ensure that it is complete and correct. If you have cited papers that have been retracted, please include the rationale for doing so in the manuscript text, or remove these references and replace them with relevant current references. Any changes to the reference list should be mentioned in the rebuttal letter that accompanies your revised manuscript. If you need to cite a retracted article, indicate the article’s retracted status in the References list and also include a citation and full reference for the retraction notice.

**Response:** The list has been reviewed and confirmed to be accurate and devoid of retracted publications.

**From the Reviewers:**

Reviewer #1: It would be useful to see the list of gene partners from the false positive list that overlap with OncoKB's Cancer gene list as a supplementary table, or provide a list of true positives as a supplementary table. If these were indeed biologically irrelevant noise, then why point out that BCOR is associated with oncogenic fusions -- isn't this an argument against using the enhanced pipeline? Perhaps focusing on a gene on the true positive list that is associated with an oncogenic signal would help to recapitulate the need for the enhanced pipeline that gives more accurate fusion calls.

**Response**: We believe the reviewer makes an excellent point with their comment and highlights a misunderstanding about the presented data. The false positives discussed in the manuscript are those that are uniquely outputted from the original pipeline but filtered out by the enhanced pipelines described in the manuscript. In other words, it represents the significant portion of fusions that were erroneously called by the original pipeline but are no longer produced by the enhanced pipelines, thus showing the utility of the enhanced pipeline. We then investigated these false positives further by comparing to the OncoKB Cancer Gene List, and identify a subset of fusion calls with biological relevance. Thus, the original pipeline produced ‘red herring’ false positives that could lead to incorrect biological and clinical interpretations, but since the enhanced pipeline filters these fusions out, it is no longer an issue for the enhanced pipelines.

While this is the intent of the section, it was not clearly stated. We have used this as an opportunity to clean up the language of that section to make this important distinction more accessible to the readers. We specifically label this class of false positives as FP_original-only_ for a convenient shorthand and easier interpretation by the reader.

Reviewer #2: The paper describes the establishment of updated open-source bioinformatics workflows for WES and RNA-seq data by the Cancer Immune Monitoring and Analysis Centers - Cancer Immunologic Data Center (CIMAC-CIDC). Previous existing pipelines were updated, benchmarked and results compared also with those of previous pipelines.

The paper provides a useful example of pipelines validation process, highlighting ground truth datasets to be utilized for this purpose as well as benchmarking measures to assess also reproducibility of pipelines. Yet the paper should and could be improved by:
1. giving more information on how the pipelines have been built and why, e.g., it was decided to favor Snakemake vs. e.g. nf-core community based pipelines;

**Response**: We thank the reviewer for this comment, as it was one that we considered during pipeline planning and development. Indeed, our team has had considerable experience and success deploying nf-core pipelines in the past. Ultimately, the decision was a practical one. The original pipelines were written with Snakemake and for ease of transition and with the desire for continuity, we chose to remain with Snakemake. The decision in no way represents a technical endorsement or critique of either (or other) workflow management tools. We have updated the text with the following information (Lines 159-164):

Snakemake was chosen as the workflow management system largely because the original pipelines also were developed with Snakemake. Other workflow management systems, such as Nextflow, were evaluated as well. While these other tools have excellent documentation, community support, and in many cases modifiable, publicly available pipelines, the desire to retain consistency and traceability between the original and enhanced pipelines led to the selection of Snakemake.

2. giving more information on how pipelines can be used and what choices are user dependent. It is not very clear how reprodicibility between users is assessed if it is not clear what choices are left to users;

**Response**: The reviewer comment highlights an important point that was missing from the manuscript but has since been corrected. The pipelines are configurable though as a rule, the configurations do not change. The CIDC is the central processing hub for the CIMAC-CIDC network and a key mandate for the CIDC is to maintain consistent, standardized processing pipelines. As such, the configurations are unchanged for all productions runs and for all runs described in the manuscript. We thank the reviewer for helping us to make this clear to the readers with the following text (Lines 122-130).

The pipelines allow user configurable modifications prior to pipeline runs. These configurations are outlined in the pipelines respective config.yaml files (WES: https://github.com/NCI-CIDC/cidc_wes2_releases/blob/main/config/config.yaml ; RNA-Seq: <https://github.com/NCI-CIDC/cidc_rnaseq2_releases/blob/main/config/config.yaml>). Cofigurable parameters include such options as output directories, cores used for the entire pipeline run and specific rules, and read trimming length. However, it is noted that these parameters are left unchanged for all productions analyses and for all validation analyses described in this study, in keeping with the prevailing CIMAC-CIDC goal of maintaining consistent, standardized data analysis.

3. improving presentation by carefully revising language (in some points of the paper entire sentences are repeated, e.g, lines 48-51, and there are some other errors) and improving figures' quality (Figures 2-4 are too small and not well readable).

**Response**: We appreciate the feedback and have used this opportunity to refine the text and figures as highlighted by the reviewer. There are numerous changes throughout the manuscript which can be found in the track changes version included in the re-submission.

We have also re-created all figures in the manuscript to for improved readability and for compliance with PLOS’s figure guidelines.
